# Exploring the relationship between extracurricular activities and stress levels among university students: A cross-sectional study

**DOI:** 10.1371/journal.pone.0329888

**Published:** 2025-08-12

**Authors:** Mona Zeidan, Rouba Ballout, Nayla Al-Akl, Samer A. Kharroubi

**Affiliations:** 1 Department of Nutrition and Food Sciences, Faculty of Agricultural and Food Sciences, American University of Beirut, Riad El Solh, Beirut, Lebanon; 2 Office of Student Affairs, American University of Beirut, Riad El Solh, Beirut, Lebanon; 3 Department of Architecture and Design, Maroun Semaan Faculty of Engineering and Architecture, American University of BeirutRiad El Solh, Beirut, Lebanon; American University of Beirut Medical Center, LEBANON

## Abstract

**Background:**

Extracurricular activities (ECA) offer students valuable opportunities to engage in nonacademic pursuits, which can be a powerful strategy for managing stress and achieving a balanced life. The present study aimed to assess the prevalence of stress among students from the American University of Beirut (AUB), and examine the association between ECA and stress levels.

**Methods:**

A cross-sectional, self-administered online survey was conducted among AUB students in Lebanon during the spring 2024 semester. Stress levels were evaluated using Cohen’s Perceived Stress Scale (PSS), a validated screening tool. The questionnaire also included general questions about students’ participation in ECA. Multinomial logistic regression analysis was applied to explore the association between ECA and stress levels, and to investigate which socio-demographic characteristics are associated with stress levels.

**Results:**

A total of 365 students completed the questionnaire. Findings showed that 71.5% of students experienced symptoms of moderate stress and 38.6% of the participants reported being involved in ECA at AUB. Our results indicated a significant association between perceived stress and participation in ECA. Students who did not participate in ECA had higher odds of experiencing stress compared to those who did (OR= 2.628, p = 0.031). Additionally, the models identified three more correlates of stress. Male students had lower odds of experiencing moderate stress than female students (OR=0.371, p = 0.008). Students who studied 10–15 hours per week had higher odds of experiencing moderate stress compared to those who studied 15 or more hours per week (OR=3.157, p = 0.043). Finally, unemployed students had higher odds of experiencing high stress compared to employed students (OR= 5.191, p < 0.001).

**Conclusions:**

This study revealed alarming stress levels among university students in Lebanon. The findings suggest that encouraging ECA participation is essential for enhancing student well-being.

## Background

During university, individuals experience significant pressures and obstacles that present various social, emotional and physical difficulties [1]. Several studies have revealed a high prevalence of stress among university students. According to Lazarus and Folkman [2], stress is considered as “a particular relationship between the person and the environment that is appraised by the person as taxing or exceeding his or her resources and endangering his or her well-being”. Several factors were found to be associated with stress levels among university students, including psychological, academic, biological, lifestyle, social, and financial factors [3]. Moreover, students often experience pressure to achieve academic success, plan for their future careers and build interpersonal relationships [4]. A survey at a Pakistani Women University in Sialkot reported that 84.4% of students experienced stress [5]. In Germany, a recent study found that 73.2% of the students at the Technical University of Munich were moderately to highly stressed [6]. Furthermore, a study conducted at University of Sharjah in UAE, found that 12.3% of the students experienced moderate stress [7]. The 2014 American College Health Association report, found that approximately half of the students experienced above-average or high level of stress within the last 12 months [8].

Although stress is not a psychiatric diagnosis, it plays a crucial role in developing psychiatric conditions such as depression and anxiety [9]. Thus, reducing stress levels can help reduce other psychological symptoms [4]. Given that university students are more prone to mental health issues compared to the general population [10]. However, universities often design extracurricular activities (ECA) to improve academic performance, as well as to offer social and emotional development [11–13]. These activities include participating in clubs like sports, social, gaming, music, and cultural clubs, volunteering and community services, and student government. Interestingly, involvement in ECA has been associated with reduced psychological stress among university students [4]. Previous studies, including high school students, have shown that ECAs are associated with positive behavior, improved grades, school completion, and characteristics that contribute to students’ success as adults [14,15]. Similarly, middle-school students involved in ECA have significantly lower dropout rates compared to those who are not [16].

Moreover, studies have highlighted the positive relationship between participation in ECA and university students’ well-being, in which students involved in ECA activities tend to have better connections and networks with their peers. In addition, volunteering and social activities play a key role in enhancing the psychological well-being in individuals and increase the sense of belonging and purpose [17]. A research conducted at a large Indian university showed that participation in ECA moderates the relationship between academic stress and coping [18]. However, there is a huge gap in the literature in Lebanon. One of the few existing studies, conducted among preclinical medical students, revealed that participating in music-related activities was correlated with lower burnout [19].

Students in Lebanon face daily challenges due to the economic and political conditions, as well as the long-lasting psychological and financial impacts resulting from the incomplete recovery from previous crises including theCOVID-19 pandemic and Beirut port explosion. Therefore, assessing stress levels among university students in Lebanon and the impact of ECA in this context is rather crucial.

The American University of Beirut (AUB) is an accredited institution that has around 8000 students and includes seven faculties: Agricultural and Food Sciences, Arts and Sciences, Engineering and Architecture (Maroun Semaan Faculty), Health Sciences, Medicine, Nursing (Rafic Hariri School), and Business (Suliman S. Olayan School). These faculties offer more than 140 programs leading to the bachelor’s, master’s, MD, and PhD degrees. At AUB, student activities involve sport clubs, social clubs, art clubs, volunteer and community services, student governments, societies and many more. These non-academic activities are not part of the regular curriculum and are usually not graded. This study aims to gain insights into AUB students’ engagement in ECA and their stress levels, providing data on the association between ECA and stress among university students in Lebanon.

Thus, the key objective of this study is to evaluate the association between ECA and stress levels among AUB students, and explore whether this relationship varies across population subgroups e.g., socio-demographic characteristics such as gender, age, major and family income.

## Methodology

### Study design and data collection

A cross-sectional online study was conducted between February and April 2024 among AUB students aged 18 and older. Sample size calculations determined that at least 384 respondents were required to estimate a prevalence of stress 50% with a 95% confidence interval and a 5% margin of error. The sample size was calculated using the World Health Organization (WHO) sample size calculator [20].

Upon receiving the Institutional Review Board (IRB) approval, an online invitation was sent out via email to a random sample of AUB students by IRB office, three reminders were sent to students. Convenience sampling was also used, the survey link was posted on social media platforms such as WhatsApp, Instagram, Twitter, LinkedIn, Facebook, in an attempt to widely reach students from different majors and class levels. Participants first reviewed the consent form (S1 Appendix) and, upon agreeing to participate, proceeded to complete the survey (S2 Appendix), which took approximately 10 minutes. Participation was entirely voluntary and anonymous. Additionally, students were encouraged to ask questions or seek further clarification before agreeing to participate. Moreover, the research team was certified by the Collaborative Institutional Training Initiative (CITI).

The study was ethically approved by the Institutional Review Board at the American University of Beirut (Protocol number: SBS-2023–0300)

### Survey format

The questionnaire consisted of three sections, developed based on previous studies [19,21,22]. The first section included socio-demographic questions, such as age, gender, household income. The second section asked students to report their participation in ECA, including the type of activities, level of involvement, and estimated number of hours spent on ECA per week. The third section used Cohen’s Perceived Stress Scale (PSS), a validated and reliable scale for assessing stress. It has demonstrated high psychometric properties and was used in various cultural contexts, including among populations that speak Arabic. [23]. The PSS consists of 10 items rated on a Likert scale from 0 (never) to 4 (very often), with reverse scoring applied to items 4, 5, 7, and 8 (i.e., 0 = 4, 1 = 3, 2 = 2, 3 = 1, 4 = 0). For each student, the assigned values were then summed. The total PSS score for each respondent ranged from 0 to 40, with scores categorized as low (0–13), moderate (14–26), and high perceived stress (27–40) [24]. The questionnaire was available in both Arabic and English to ensure accessibility for all AUB students.

To test the questionnaire’s clarity, twenty students participated in a pilot study. Data gathered from the pilot test were not included in the final analysis.

### Statistical analysis

After checking for completeness, the data was analyzed using the Statistical Package for the Social Sciences (SPSS) version 29.0. Descriptive statistics for continuous variables were reported as means and standard deviations (SD); whereas frequencies and proportions were reported for categorical variables. Chi-square (χ^2^) test was used to study the associations between categorical variables. Multinomial logistic regression analysis was applied to explore the association between ECA and stress levels, and to investigate which socio-demographic characteristics are associated with stress levels. In this analysis, stress levels with the three subcategories (low, moderate, and high) served as the dependent variable, whereas sociodemographic factors (for instance age, education, and income) and participation in ECA were the independent variables. The multiple regression models included only variables that demonstrated statistical significance in the simple analysis. Results from the multinomial logistic regression analyses were expressed as odds ratios (OR) with 95% confidence intervals (CI). A p-value of less than 0.05 was considered statistically significant for all analyses.

## Results

### Sociodemographic characteristics and its association with perceived stress (PS) levels

A total of 384 AUB students participated in the online survey, with 365 providing complete responses, resulting in a 95% completion rate. Table 1 summarizes the sociodemographic characteristics of the study population. More than half of the students (55.1%) were between 18 and 20 years old, with junior students (third year of undergraduate study) constituting the highest percentage (47.4%). Almost two-thirds of the participants were female (67.1%). The majority of the students (75.1%) lived with their families. Most students were Lebanese (92.6%) and full-time students (90.4%), with 61.5% enrolled in non-health related majors. Nearly half of the students (49.9%) reported a monthly household income of $1000 or more. Approximately 36% of the students spent 5–10 hours on study per week. Almost half of the participants (53.15%) were employed, and among the 194 employed students, about 26% worked 1–5 hours per week.

**Table 1:** Socio-demographic characteristics of AUB students in the study sample (n = 365) (Spring 2024-Lebanon).

Characteristics		n (%)	Low PS	Moderate PS	High PS	p-value
Age (n = 365)	18-20	201 (55.1)	18 (9.0)	147 (73.1)	36 (17.9)	0.371
	21-23	90 (24.7)	12 (13.3)	58 (64.4)	20 (22.2)	
	24-26	39 (10.7)	4 (10.3)	27 (69.2)	8(20.5)	
	27+	35 (9.6)	4 (11.4)	29 (82.9)	2 (5.7)	
Gender (n = 359)	Male	118 (32.9)	22 (18.6)	83 (70.3)	13 (11.0)	<0.001
	Female	241 (67.1)	15 (6.2)	175 (72.6)	51 (21.2)	
Nationality (n = 365)	Lebanese	338 (92.6)	35 (10.4)	242 (71.6)	61 (18.0)	0.989
	Non-Lebanese	27 (7.4)	3 (11.1)	19 (70.4)	5 (18.5)	
Monthly income of household (USD) (n = 351)	<500	40 (11.4)	1 (2.5)	33 (82.5)	6 (15)	0.278
	[500-800]	70 (19.9)	7 (10.0)	52 (74.3)	11 (15.7)	
	[800-1000]	66 (18.8)	5 (7.6)	47 (71.2)	14 (21.2)	
	≥1000	175 (49.9)	25 (14.3)	116 (66.3)	34 (19.4)	
Major (n = 364)	Health related	140 (38.5)	13 (9.3)	103 (73.6)	24 (17.1)	0.916
	Non-health related	224 (61.5)	25 (11.2)	157 (70.1)	42 (18.8)	
Full-time Student (n = 365)	Yes	330 (90.4)	36 (10.9)	234 (70.9)	60 (18.2)	0.603
	No	35 (9.6)	2 (5.7)	27 (77.1)	6 (17.1)	
Time spent on Study (per week) (n = 365)	[1–5]hours	66 (18.1)	7 (10.6)	49 (74.2)	10 (15.2)	0.306
	[5–10]hours	131 (35.9)	11 (8.4)	94 (71.8)	26 (19.8)	
	[10–15]hours	89 (24.4)	6 (6.7)	65 (73.0)	18 (20.2)	
	≥15 hours	79 (21.6)	14 (17.7)	53 (67.1)	12 (15.2)	
Employed (n = 365)	Yes	194 (53.15)	26 (13.4)	148 (76.3)	20 (10.3)	<0.001
	No	171 (46.85)	12 (7.0)	113 (66.1)	46 (26.9)	
If employed, hours spent on work per week (n = 194)	[1–5]hours	94 (25.8)	9 (9.6)	71 (75.5)	14 (14.9)	0.245
	[5–10]hours	47 (12.9)	9 (19.1)	37 (78.7)	1 (2.1)	
	[10–15]hours	19 (5.2)	3 (15.8)	15 (78.9)	1 (5.3)	
	≥15 hours	34 (9.3)	5 (14.7)	25 (73.5)	4 (11.8)	
Residency during university term time (n = 365)	With family	274 (75.1)	25 (9.1)	195 (71.2)	54 (19.7)	0.313
	With roommates	61 (16.7)	10 (16.4)	42 (68.9)	9 (14.8)	
	Alone	30 (8.2)	3 (10.0)	24 (80.0)	3 (10.0)	
Stage of Study (n = 365)	Sophomore	107 (29.3)	11 (10.3)	80 (74.8)	16 (15.0)	0.829
	Junior	173 (47.4)	18 (10.4)	125 (72.3)	30 (17.3)	
	Senior	53 (14.5)	5 (9.4)	35 (66.0)	13 (24.5)	
	Other (master, PhD)	32 (8.8)	4 (125)	21 (65.6)	7 (21.9)	

As shown in Table 1, significant differences were observed in the perceived stress levels of AUB students based on their gender (p < 0.001) and employment status (p < 0.001).

### Extracurricular activities (ECA)

The results in Table 2 revealed that almost two-thirds of the participants (61.4%) were not involved in ECA during their study period, while 38.6% were involved in either single or multiple activities. The most ECA were student societies (52.5%), sport clubs (34.7%), and social club (26.9%). Among those who participated in ECA (n = 141), more than half (52.5%) spent 1 to 5 hours per week on these activities. A significant majority of students (84.4%) reported making friends and expanding their social network as a result of their ECA engagement. About 28.1% of those engaged in ECA felt that their involvement significantly reduced their stress levels, and 37.4% considered that engagement in ECA had a positive effect on their studies. A small portion of the participants (13.4%) faced certain challenges such as time management due to their ECA engagement. Additionally, 55.7% of the students agreed that the ECA system at AUB is easy to use, while less than half (40.9%) reported being fully informed on how to use the ECA system.

**Table 2:** Participation in ECA of AUB students in the study sample (n = 365) (Spring 2024-Lebanon).

ECA participation		n (%)
Currently involved in any extracurricular activities (ECA) at the AUB (n = 365)	Yes	141 (38.6)
	No	224 (61.4)
Activities in which students engaged since the start of their university years (Choose all that apply)1	n = 141	
	Sport clubs	49 (34.7)
	Student societies	74 (52.5)
	Social club	38 (26.9)
	Book club	1 (0.7)
	Gaming club	2 (1.4)
	Artist club	5 (3.5)
	Music club	8 (5.7)
	Cultural clubs	8 (5.7)
	Volunteer and community services	54 (38.3)
	Student government	8 (5.7)
	Other	5 (3.5)
Time spent on ECA per week:	[1–5]hours	73 (52.5)
	[5–10]hours	38 (27.3)
	[10–15]hours	15 (10.8)
	≥15 hours	13 (9.4)
Students made new friends or expanded their social network through participation in ECA	Yes	114 (84.4)
	No	21 (15.6)
The ECA system at AUB is easy to use (1 = strongly disagree, 5 = strongly agree)	Strongly Disagree	8 (5.7)
	Disagree	11 (7.9)
	Neutral	43 (30.7)
	Agree	48 (34.3)
	Strongly Agree	30 (21.4)
I am fully informed on how to use the ECA system (1 = strongly disagree, 5 = strongly agree)	Strongly Disagree	13 (9.5)
	Disagree	27 (19.7)
	Neutral	41 (29.9)
	Agree	29 (21.2)
	Strongly Agree	27 (19.7)
Engagement in ECA has reduced students’ stress level	Not at all	21 (15.1)
	A little	27 (19.4)
	Moderately	37 (26.6)
	Quite a bit	39 (28.1)
	Extremely	15 (10.8)
Engagement in ECA has affected students’ study	Yes, positively	52 (37.4)
	Yes, negatively	18 (12.9)
	No	44 (31.7)
	Don’t know	25 (18)
Challenges or difficulties faced because of the engagement in ECA	Yes	18 (13.4)
	No	116 (86.6)

^1^Participants can choose several activities. 68 (48.2%) students engaged in one ECA, and 73 (51.8%) in more than one ECA.

### Cohen’s Perceived Stress Scale (PSS)

**Fig 1:**
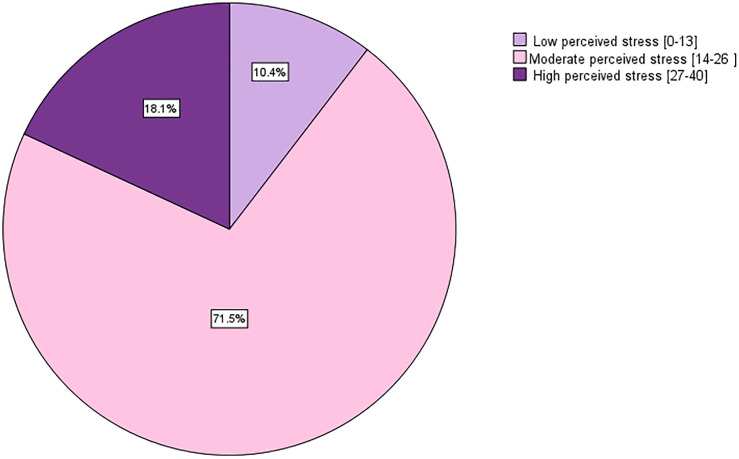
Perceived stress of AUB students in the study sample (n = 365) (Spring 2024-Lebanon).

Among the study sample (Fig 1), the majority of the students (71.5%) exhibited moderate perceived stress level, 18.1% exhibited high perceived stress level, and 10.4% exhibited low perceived stress level.

### Multinomial logistic regression analysis

Simple multinomial logistic analysis revealed that four predictors were significantly associated with the perceived stress level among university students (Table 3). Gender was one of the predictors, with males being less likely to exhibit moderate (OR = 0.323, p = 0.002) and high stress levels (OR = 0.174, p < 0.001) than females. Other significant predictors comprised the time spent on study per week (Moderate perceived stress: OR = 2.862, p = 0.044; High perceived stress: OR = 3.500, p = 0.041), unemployment status (OR = 4.983, p < 0.001), and the involvement in ECA (Moderate perceived stress: OR = 2.089, p = 0.036; High perceived stress: OR = 2.309, p = 0.044). These predictors remained significant in the multiple analysis. For instance, males had lower odds of experiencing moderate stress (OR = 0.371, p = 0.008) and high stress (OR = 0.238, p = 0.003) compared to females. In addition, students who studied 10–15 hours per week had higher odds of exhibiting moderate stress compared to those who studied 15 or more hours per week (OR = 3.157, p = 0.043). Unemployed participants had higher odds of experiencing high stress compared to employed (OR = 5.191, p = < 0.001). Finally, students not engaged in ECA were more likely to present high stress level than those who were involved in ECA (OR=2.628, p = 0.031).

**Table 3:** Simple and Multiple Logistic Regression Analysis for the predictors of stress among AUB students in the study sample (n = 365) (Spring 2024-Lebanon).

Characteristics		Simple Regression OR, (95% CI), p-Value	Multiple Regression OR, (95% CI), p-Value
**Age**	**Moderate**		
	18-20	-	
	21-23	0.592 (0.268, 1.306); 0.194	
	24-26	0.827 (0.259, 2.633); 0.747	
	27+	0.888 (0.280, 2.816); 0.840	
	**High**		
	18-20	-	
	21-23	0.833 (0.335, 2.075); 0.695	
	24-26	1.000 (0.265, 3.769); 1.000	
	27+	0.250 (0.042, 1.496); 0.129	
**Gender**	**Moderate**		
	Male	**0.323 (0.160, 0.655); 0.002**	**0.371 (0.179, 0.767); 0.008**
	Female	-	-
	**High**		
	Male	**0.174 (0.071, 0.425); <0.001**	**0.238 (0.094, 0.605); 0.003**
	Female	-	-
**Nationality**	**Moderate**		
	Lebanese	1.092 (0.307, 3.880); 0.892	
	Non-Lebanese	-	
	**High**		
	Lebanese	1.046 (0.236, 4.642); 0.953	
	Non-Lebanese	-	
**Monthly income of household (USD)**	**Moderate**		
	<500	7.112 (0.929, 54.469); 0.059	
	[500-800]	1.601 (0.651, 3.937); 0.305	
	[800-1000]	2.026 (0.732, 5.608); 0.174	
	≥1000	-	
	**High**		
	<500	4.412 (0.499, 38.992); 0.182	
	[500-800]	1.155 (0.393, 3.400); 0.793	
	[800-1000]	2.059 (0.656, 6.465); 0.216	
	≥1000	-	
**Major**	**Moderate**		
	Health related	-	
	Non-health related	0.793 (0.388, 1.620); 0.524	
	**High**		
	Health related	-	
	Non-health related	0.910 (0.394, 2.102); 0.825	
**Full-time student**	**Moderate**		
	Yes	0.481 (0110, 2.112); 0.333	
	No	-	
	**High**		
	Yes	0.556 (0.106, 2.901); 0.486	
	No	-	
**Time spent on Study (per week)**	**Moderate**		
	[1-5] hours	1.849 (0.689, 4.961); 0.222	1.757 (0.635, 4.866); 0.278
	[5-10] hours	2.257 (0.957, 5.326); 0.063	2.112 (0.867, 5.142); 0.100
	[10-15] hours	**2.862 (1.029, 7.958); 0.044**	**3.157 (1.038, 9.601); 0.043**
	≥15 hours	-	-
	**High**		
	[1-5] hours	1.667 (0.484, 5.736); 0.418	1.700 (0.463, 6.235); 0.424
	[5-10] hours	2.758 (0.970, 7.839); 0.057	2.517 (0.826, 7.672); 0.104
	[10-15] hours	**3.500 (1.051, 11.660); 0.041**	3.618 (0.968, 13.516); 0.056
	≥15 hours	-	-
**Employed**	**Moderate**		
	Yes	-	-
	No	1.654 (0.800, 3.421); 0.174	1.699 (0.776, 3.717); 0.185
	**High**		
	Yes	-	-
	No	**4.983 (2.104, 11.803); <0.001**	**5.191 (2.049, 13.153); <0.001**
**Residency during university time**	**Moderate**		
	With Family	-	
	With Roommates	0.538 (0.241, 1.205); 0.132	
	Alone	1.026 (0.288, 3.654); 0.969	
	**High**		
	With Family	-	
	With Roommates	0.417 (0.151, 1.153); 0.092	
	Alone	0.463 (0.087, 2.457); 0.366	
**Stage of Study**	**Moderate**		
	Sophomore	-	
	Junior	0.955 (0.429, 2.127); 0.910	
	Senior	0.962 (0.311, 2.977); 0.947	
	Other (Master, PhD)	0.722 (0.209, 2.497); 0.607	
	**High**		
	Sophomore	-	
	Junior	1.146 (0.437, 3.007); 0.782	
	Senior	1.788 (0.494, 6.466); 0.376	
	Other (Master, PhD)	1.203 (0.283, 5.122); 0.802	
**Involvement in ECA**	**Moderate**		
	No	**2.089 (1.051, 4.151); 0.036**	2.034 (0.992, 4.172); 0.053
	Yes	-	-
	**High**		
	No	**2.309 (1.022, 5.221); 0.044**	**2.628 (1.090, 6.334); 0.031**
	Yes	-	-

## Discussion

Several studies in Lebanon have addressed the mental health issues, stress levels and their effects on university students [25,26]. However, to the best of our knowledge, few studies have focused on the factors that help reduce stress and improve mental health among university students. This study provides significant insights into the relationship between extracurricular involvement and perceived stress levels of students at AUB.

The results of our study indicate that a significant proportion of students at AUB experienced moderate stress levels (71.5%), while a smaller proportion reported high perceived stress levels (18.1%) and low perceived stress levels (10.4%). These findings differ from a study in Pakistan that indicated a lower prevalence of moderate perceived stress levels among university students [5]. Conversely, several studies demonstrated results aligning with our findings in which the highest proportion of students experienced moderate stress levels [27,28]. One of these studies, conducted in Oman, revealed that almost three-quarters of the students suffered from moderate stress (75.1%) and 13.5% reported severe stressors [27]. In Lebanon, several studies have assessed stress levels among university student using the perceived stress scale (PSS)., One study, which included 261 students from three different universities revealed slightly higher moderate stress level among students (75.1%), and a lower level of high stress (13.4%) [26]. Another study included 427 students demonstrated lower levels of moderate stress (41%) and high stress (16.1) [29]. Additionally, a study performed on 165 pre-clinical medical students, using the General Health Questionnaire (GHQ), found that 62% of the students suffered from stress [19]. The high prevalence of stress among university students in Lebanon could be attributed to several factors, including the economic crisis, political situation, and the lingering impacts of the COVID-19 pandemic [30].

The main objective of this study was to investigate the relationship between extracurricular activities and stress levels among students at AUB. Our findings indicated that students not engaged in ECA were more likely to experience high stress levels compared to those who were involved. On the contrary, previous studies, conducted on undergraduate students in Turkey and medical students in Saudi Arabia, demonstrated no significant difference in stress and burnout levels between students involved in ECA and those who were not [22,31]. This discrepancy could be due to the variety of extracurricular activities available at different universities. On the other hand, our results align with several other findings that found a negative correlation between involvement in ECAs and stress levels, indicating that engagement in ECAs helps reduce stress levels [4,21,32]. This could be due to the fact that students involved in ECA build friendships and develop their social network, thus enhancing their social bonds and reducing feelings of isolation [4]. Besides, participation in physical activities enhances the release of endorphin, which are natural stress relievers [33]. Furthermore, involvement in ECA can help students distract themselves from academic pressure.

Furthermore, various stress factors among the participants were also addressed. Our findings identified three predictors associated with stress levels among the students: gender, time spent on study, and employment. Gender differences revealed that females experienced more stress than males, contradicting previous studies that found no statistically significant difference in stress levels between males and females among college students in Eritrea and China and university students in Pakistan [28,34,35]. However, studies in Turkey and Lebanon align with our results, indicating higher stress levels among females [29,36]. The significant difference in stress levels between males and females can be attributed to several factors. Generally, females are known to exert more effort in their studies than males, which can be a major cause of stress [37]. The pressure to perform and the fear of failure are also critical risk factors for stress. Moreover, a study conducted in the US suggested that females may experience higher stress due to academics and relationships, whereas males report greater stress related to financial issues [38]. Additionally, a review on the differences in anxiety between males and females indicated that psychosocial and biological factors play a major role [39]. Culturally, women in many societies tend to express their emotions more openly than men, which may contribute to the observed differences in stress levels. A study published in the *Journal of Personality and Social Psychology* found that women are generally more emotionally expressive, whereas men exhibit stronger emotional responses to specific stimuli, such as anger and positive events [40]. Consequently, women’s greater ability to articulate their feelings of stress may partly explain the differences observed in our study.

Additionally, our findings revealed that students who study 10–15 hours per week are more likely to experience moderate stress levels compared to those who study more than 15 hours. In contrast, a study on children in China revealed that time spent on homework is a positive predictor of stress symptoms, leading to depression [41]. This discrepancy could be due to the fact that students who study fewer hours may feel their study time is insufficient, leading to increased stress from believing they should have studied more. Additionally, the difference could be attributed to variations in curriculums between Lebanon and China, which may require different amounts of study time.

Regarding employment, our findings revealed that unemployed students were more likely to experience higher stress levels compared to employed students. These findings align with a study in Ontario, Canada, which demonstrated significant differences in psychosocial experiences between unemployed and employed students [42]. This could be because employed students feel more financially stable than unemployed students, which may experience financial stress [42]. Additionally, the majority of employed students in this study work between 1–5 hours per week, suggesting they do not have full-time jobs, thus avoiding significant time management conflicts. Our study did not identify whether the students have full scholarships, financial aid, or cover their entire tuition fees. Therefore, among unemployed students, there may be those with full scholarships who experience stress while maintaining a high GPA.

Several limitations may have influenced our findings. First, the cross-sectional design of the study allows for the identification of associations but does not allow for establishing causal relationships. Second, the use of a non-random sampling method may have introduced selection bias, as students experiencing higher stress levels may have been more inclined to participate in the study. Third, the study was conducted among AUB students which does not allow for the generalizability of the results to students from other universities in Lebanon and other countries. Additionally, the data was based on self-reports responses, which may be subject to reporting or social desirability bias. Despite these limitations, our findings indicate that engagement in ECA helps lower stress level among AUB students. This provides universities valuable insights into the importance of offering extracurricular activities to students due to their mental health benefits. Moreover, students will be able to understand the importance of these activities in improving their mental and physical health.

## Conclusion

The present study investigated the relationship between stress levels and participation in ECA among students at the American University of Beirut (AUB). The study revealed alarming stress levels, highlighting the unique challenges faced by university students in Lebanon. However, engagement in ECA was associated with lower stress levels. Consequently, the Office of Student Affairs has evidence and data-driven insights on the importance of promoting participation in ECA to enhance students’ well-being. Moreover, given that AUB is among the most reputable universities in the MENA region, results suggest that students in other universities may experience even higher level of stress, especially if there is a lack of well-structured ECA program and mental health support system. Thus, there is a need for future research across all universities in Lebanon, particularly those with fewer resources. This could help in identifying disparities and guiding national initiatives to enhance the well-being of students. While this study highlights the importance of ECA in reducing stress levels, it is crucial to understand that different types of ECAs have different psychological effects, with some activities being more effective than others. Future studies should thus examine the various impacts that different forms of ECA have on students’ mental health.

## Supporting information

S1 Appendix Informed Consent Form. (DOCX)

S2 Appendix Questionnaire. (DOCX)

S3 Appendix Data of the study. (XLSX)

## References

[1] Rodgers LS, Tennison LR. A preliminary assessment of adjustment disorder among first-year college students. Arch Psychiatr Nurs. 2009;23(3):220–30. doi: 10.1016/j.apnu.2008.05.007 19446777

[2] Lazarus RS, Folkman S. Stress: appraisal and coping. Springer. 2013.

[3] Mofatteh M. Risk factors associated with stress, anxiety, and depression among university undergraduate students. AIMS Public Health. 2020;8(1):36–65. doi: 10.3934/publichealth.2021004 33575406 PMC7870388

[4] Wang T, Yao Z, Liu Q, Zhao J, Wang X, Wong JP-H, et al. The Mediating Effect of Stress between Extracurricular Activities and Suicidal Ideation in Chinese College Students. Int J Environ Res Public Health. 2023;20(4):3105. doi: 10.3390/ijerph20043105 36833799 PMC9963993

[5] Asif S, Mudassar A, Shahzad TZ, Raouf M, Pervaiz T. Frequency of depression, anxiety and stress among university students. Pakistan Journal of Medical Sciences. 2020;36(5):971. 32704273 10.12669/pjms.36.5.1873 PMC7372668

[6] Olson N, Oberhoffer-Fritz R, Reiner B, Schulz T. Stress, student burnout and study engagement - a cross-sectional comparison of university students of different academic subjects. BMC Psychol. 2025;13(1):293. doi: 10.1186/s40359-025-02602-6 40128867 PMC11931745

[7] Al-Garni AM, Shati AA, Almonawar NA, Alamri GM, Alasmre LA, Saad TN, et al. Prevalence of depression, anxiety, and stress among students enrolled at King Khalid University: a cross-sectional study. BMC Public Health. 2025;25(1):354. doi: 10.1186/s12889-025-21277-7 39875847 PMC11773868

[8] American College Health Association. American College Health Association-National College Health Assessment II: Reference group executive summary. (accessed Spring 2014). https://www.acha.org

[9] Rois R, Ray M, Rahman A, Roy SK. Prevalence and predicting factors of perceived stress among Bangladeshi university students using machine learning algorithms. J Health Popul Nutr. 2021;40(1):50. doi: 10.1186/s41043-021-00276-5 34838133 PMC8627029

[10] Lee RA, Jung ME. Evaluation of an mHealth App (DeStressify) on University Students’ Mental Health: Pilot Trial. JMIR Ment Health. 2018;5(1):e2. doi: 10.2196/mental.8324 29362209 PMC5801522

[11] Buckley P, Lee P. The impact of extra-curricular activity on the student experience. Active Learning in Higher Education. 2018;22(1):37–48. doi: 10.1177/1469787418808988

[12] Christison C. The benefits of participating in extracurricular activities. BU Journal of Graduate Studies in Education. 2013;5(2):17–20.

[13] Joseph NA. Exploring the relationship between extracurricular participation & probability of employment for high school graduates. Georgetown University; 2009.

[14] Shaffer ML. Impacting Student Motivation: Reasons for Not Eliminating Extracurricular Activities. Journal of Physical Education, Recreation & Dance. 2019;90(7):8–14. doi: 10.1080/07303084.2019.1637308

[15] Massoni E. Positive effects of extra curricular activities on students. Essai. 2011;9(1):27.

[16] Mahoney JL, Cairns RB. Do extracurricular activities protect against early school dropout?. Dev Psychol. 1997;33(2):241–53. doi: 10.1037//0012-1649.33.2.241 9147833

[17] Mehmood U, et al. Relationship between curricular activities and stress management at college level. 2024.

[18] Mukesh HV, Acharya V, Pillai R. Are extracurricular activities stress busters to enhance students’ well-being and academic performance? Evidence from a natural experiment. Journal of Applied Research in Higher Education. 2023;15(1):152–68.

[19] Fares J, Saadeddin Z, Al Tabosh H, Aridi H, El Mouhayyar C, Koleilat MK, et al. Extracurricular activities associated with stress and burnout in preclinical medical students. J Epidemiol Glob Health. 2016;6(3):177–85. doi: 10.1016/j.jegh.2015.10.003 26644345 PMC7320478

[20] Sample Size Calculator. https://cdn.who.int/media/docs/default-source/ncds/ncd-surveillance/steps/sample-size-calculator.xls?sfvrsn=ee1f4ae8_2

[21] Finnerty R, Marshall SA, Imbault C, Trainor LJ. Extra-Curricular Activities and Well-Being: Results From a Survey of Undergraduate University Students During COVID-19 Lockdown Restrictions. Front Psychol. 2021;12:647402. doi: 10.3389/fpsyg.2021.647402 34262502 PMC8274476

[22] Civitci A. Perceived Stress and Life Satisfaction in College Students: Belonging and Extracurricular Participation as Moderators. Procedia - Social and Behavioral Sciences. 2015;205:271–81. doi: 10.1016/j.sbspro.2015.09.077

[23] Chaaya M, Osman H, Naassan G, Mahfoud Z. Validation of the Arabic version of the Cohen Perceived Stress Scale (PSS-10) among pregnant and postpartum women. BMC Psychiatry. 2010;10:111. doi: 10.1186/1471-244X-10-111 21159169 PMC3016315

[24] Malik M, Javed S. Perceived stress among university students in Oman during COVID-19-induced e-learning. Middle East Current Psychiatry. 2021;28(1):49.

[25] Halat DH, et al. Exploring the effects of health behaviors and mental health on students’ academic achievement: a cross-sectional study on lebanese university students. BMC Public Health. 2023;23. 10.1186/s12889-023-16184-8 PMC10291759 37365573

[26] Kharroubi SA, Al-Akl N, Chamate S-J, Abou Omar T, Ballout R. Assessing the Relationship between Physical Health, Mental Health and Students’ Success among Universities in Lebanon: A Cross-Sectional Study. Int J Environ Res Public Health. 2024;21(5):597. doi: 10.3390/ijerph21050597 38791811 PMC11121208

[27] Alkhawaldeh A, et al. Stress factors, stress levels, and coping mechanisms among university students. The Scientific World Journal. 2023;2023. 10.1155/2023/2026971 PMC10325878 37426577

[28] Yikealo D, Tareke W, Karvinen I. The level of stress among college students: A case in the college of education, Eritrea Institute of Technology. Open Science Journal. 2018;3(4).

[29] Taleb R, et al. Stress and coping strategies among undergraduate health professions students: A cross-sectional study from a university in Lebanon. BAU Journal-Health and Wellbeing. 2020;3(1):2.

[30] Farran N. Mental health in Lebanon: Tomorrow’s silent epidemic. Ment Health Prev. 2021;24:200218. doi: 10.1016/j.mhp.2021.200218 34660191 PMC8503814

[31] Almalki SA, Almojali AI, Alothman AS, Masuadi EM, Alaqeel MK. Burnout and its association with extracurricular activities among medical students in Saudi Arabia. Int J Med Educ. 2017;8:144–50. doi: 10.5116/ijme.58e3.ca8a 28454079 PMC5420457

[32] Guilmette M, Mulvihill K, Villemaire-Krajden R, Barker ET. Past and present participation in extracurricular activities is associated with adaptive self-regulation of goals, academic success, and emotional wellbeing among university students. Learning and Individual Differences. 2019;73:8–15. doi: 10.1016/j.lindif.2019.04.006

[33] Pangkahila EA, Adiputra N, Pangkahila W, Yasa IWPS. Balanced physical exercise increase physical fitness, optimize endorphin levels, and decrease malondialdehyde levels. Bali Medical Journal. 2016;5(3):493–6.

[34] Gao W, Ping S, Liu X. Gender differences in depression, anxiety, and stress among college students: A longitudinal study from China. J Affect Disord. 2020;263:292–300. doi: 10.1016/j.jad.2019.11.121 31818792

[35] Talib N, Zia-ur-Rehman M. Academic performance and perceived stress among university students. Educational Research and Reviews. 2012;7(5):127.

[36] Sayıner B. Stress level of university students. İstanbul Ticaret Üniversitesi Fen Bilimleri Dergisi. 2006;5(10):23–34.

[37] Özen NS, Ercan İ, İrgil E, Sığırlı DS. Anxiety prevalence and affecting factors among university students. 2010. 10.1177/1010539509352803 20032042

[38] Gefen DR, Fish MC. Gender differences in stress and coping in first-year college students. Journal of College Orientation, Transition, and Retention. 2012;19(2).

[39] Farhane-Medina NZ, Luque B, Tabernero C, Castillo-Mayén R. Factors associated with gender and sex differences in anxiety prevalence and comorbidity: A systematic review. Sci Prog. 2022;105(4):368504221135469. doi: 10.1177/00368504221135469 36373774 PMC10450496

[40] Kring AM, Gordon AH. Sex differences in emotion: expression, experience, and physiology. J Pers Soc Psychol. 1998;74(3):686–703. doi: 10.1037//0022-3514.74.3.686 9523412

[41] Xiao P, Zhu K, Liu Q, Xie X, Jiang Q, Feng Y, et al. Association between developmental dyslexia and anxiety/depressive symptoms among children in China: The chain mediating of time spent on homework and stress. J Affect Disord. 2022;297:495–501. doi: 10.1016/j.jad.2021.10.120 34743962

[42] Jackson T. Differences in psychosocial experiences of employed, unemployed, and student samples of young adults. J Psychol. 1999;133(1):49–60. doi: 10.1080/00223989909599721 10022077

