# Supplementary material for: Exploring the relationship between extracurricular activities and stress levels among university students: A cross-sectional study
Source: PLoS One. 2025 Aug 12;20(8):e0329888. doi: 10.1371/journal.pone.0329888 (PMC12342330; doi:10.1371/journal.pone.0329888)
Supplement: S1 Appendix — (DOCX) [file pone.0329888.s001.docx]

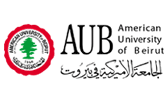


**Appendix A: Informed Consent Form**

**Title of Research Study:** Exploring the Relationship Between Extracurricular Activities and Stress Levels among University Students: A Cross-Sectional Study

**Principal Investigator:** Dr. Samer Kharroubi-Department of Nutrition and Food Sciences, American University of Beirut.

**INTRODUCTION**

You are invited to participate in a research study conducted by the Department of Nutrition and Food Sciences at the American University of Beirut (AUB). Please take time to read the information below carefully before agreeing that you participate in the study, to understand the purpose, actions, benefits and risks related to your participation in the project. Please feel free to ask any questions if you need more information or clarification about what is stated in this form and the study as a whole.

**PURPOSE**

The purpose of this research study is to investigate the associations between the participation in extracurricular activities and student’s stress levels. We will first ask you about some socio-demographic characteristics such as household income, area of residency, and employment status. We will also ask you some questions about your participation in extracurricular activities such as type of activity, frequency… Finally, the study will collect information on your rating of perceived stress

**PROCEDURE**

A representative sample of 384 AUB students aged 18 years old or above, literate and with no physical or mental incapacity, that would prevent reading and understanding required in the valuation tasks, will be recruited for this study. Recruitment of the study participants will take place through an online invitation that will be sent out via **emails** to a random sample of **AUB students** starting spring 2024 and upon receiving IRB approval. The survey link will also be posted on social media platforms related to the university and on research team members’ personal platforms such as WhatsApp, Instagram, Twitter, LinkedIn, Facebook, in an attempt to widely reach students from different majors (arts & sciences, agriculture, public health, engineering, business) and class levels (freshman, sophomore, etc.). The research team will contact group admins to ensure their approval to share survey link on their platform.

Email invitations will be sent to students by HRPP/IRB office. No email lists of AUB students will be shared with the research team.

The invitation will include the link to the survey and consent form.

The survey will require around 10 minutes of your time. Below is a description of the sections if you decide to participate in the study:

First, you will be asked about some socio-demographic characteristics (age, gender, area of residency…).

Next, you will be asked some questions that help to assess your participation in extracurricular activities.

Finally, you will be asked to complete the Perceived Stress Scale (PSS), which is validated screening tool that assess stress levels.

A referral list with relevant NGOs and the counseling services that are available at AUB will be provided to participating students.

Participants will also be provided with an information sheet about activities available at AUB and a tip sheet on how to cope with stress.

**RISK, DISCOMFORTS AND BENEFITS**

There are no direct benefits and no anticipated risks associated with the participation in this study.

Also note that your participation is entirely on a voluntary basis and you have the right to withdraw your consent or discontinue participation at any time without penalty. You also have the option to skip any question. Withdrawal or refusal to participate will involve no penalty or loss of benefits.

**CONFIDENTIALITY**

If you agree to participate in this research study, the information will be kept confidential. The questionnaire is anonymous and there will not be any collection of personal identifiers. We will assign a code to identify you in our records instead of using your name. Your records will be monitored and audited without violating your confidentiality. Your individual privacy will be maintained in all published research and reports resulting from this study.

Only the members of the research group will have access to the questionnaires that will be used for research purposes only. The filled questionnaires will be locked and secured in a password protected pc that could only be accessed by the investigator. Also, your records will be monitored and may be audited by representatives of the Institutional Research Board (IRB) who will make sure the study is conducted properly and that your rights are protected while assuring confidentiality.

**CONTACT INFORMATION AND QUESTIONS**

A soft copy of this consent form will be left with you and if you have any questions or concerns about the research, you may contact:

Dr. Samer A Kharroubi, Faculty of Agricultural & Food Sciences-AUB

**Tel:** 961-1-350000 (Ext 4541) **E-mail:** sk157@aub.edu.lb

If you have any questions, concerns or complaints about your rights as a participant in this research, you can contact the following office at AUB:

Social & Behavioral Sciences Institutional Review Board

**Address:** American University of Beirut; Riad El Solh, Beirut 1107 2020, Lebanon

**Tel:** 00961 1 374374, ext: 5445 **Email:** [irb@aub.edu.lb](mailto:irb@aub.edu.lb)

**ACCESS TO THE SURVEY**

If after reading the consent document and having your questions answered, you voluntarily

agree to take part in the study, you can access the survey by answering the questions below.
