# Supplementary material for: Exploring the relationship between extracurricular activities and stress levels among university students: A cross-sectional study
Source: PLoS One. 2025 Aug 12;20(8):e0329888. doi: 10.1371/journal.pone.0329888 (PMC12342330; doi:10.1371/journal.pone.0329888)
Supplement: S2 Appendix — (DOCX) [file pone.0329888.s002.docx]

**Appendix B: Questionnaire**

**Socio-demographics**

**Are you a student at the American University of Beirut?**

- Yes
- No

**Age:**

- 18-20
- 21-23
- 24-26
- 27+

**Sex:**

- Male
- Female
- Prefer not to answer

**Nationality:**

- Lebanese
- Non-Lebanese

**What is the monthly income of your household?**

- <500 USD
- 500-800 USD
- 800-1000 USD
- ≥ 1000 USD

**Major:**

- Business
- Engineering, Computer science
- Health-related (Nutrition, Medical Lab, Public Health, Medical Sciences)
- Arts
- Sciences (Biology, Chemistry, Physics)
- Other: -------

**Are you a full-time student?**

- Yes
- No

**Time spent on study (per week):**

- 1-5 hours
- 5-10 hours
- 10-15 hours
- ≥15 hours

**Are you employed?**

- Yes
- No

**If you are employed, how many hours do you currently work per week?**

- 1-5 hours
- 5-10 hours
- 10-15 hours
- ≥15 hours

**Where do you live during university term time?**

- With family
- With room-mates
- Alone
- Other: --------

**Stage of study:**

- Sophomore
- Junior
- Senior
- Other (Master, Phd)

**Extracurricular activities:**

**Are you currently involved in any extracurricular activities (ECA) at the AUB?**

- Yes
- No

**Which of the following activities have you engaged in since the start of your university years? (Choose all that apply)**

- Sport clubs
- Student societies
- Social club
- Book club
- Gaming club
- Artist club
- Music club
- Cultural clubs
- Volunteer and community services
- Student government
- Other: ----------------

**Time spent on ECA per week:**

- 1-5 hours
- 5-10 hours
- 10-15 hours
- ≥15 hours

**Have you made new friends or expanded your social network through your participation in ECA?**

- Yes
- No

**The ECA system at AUB is easy to use (1= strongly disagree, 5=strongly agree)**

- 1
- 2
- 3
- 4
- 5

**I am fully informed on how to use the ECA system** **(1= strongly disagree, 5=strongly agree)**

- 1
- 2
- 3
- 4
- 5

**Do you feel that your engagement in ECA have reduced your stress level?**

- Not at all
- A little
- Moderately
- Quite a bit
- Extremely

**Do you feel that your engagement in ECA have affected your study?**

- Yes, positively
- Yes, negatively
- No
- Don’t know

**Have you faced any challenges or difficulties as a result of your engagement in ECA?**

- Yes
- No

**If yes please specify: ----------**

**Perceived Stress Scale (PSS):**

| **In the last month, how often have you…** | **Never**  **0** | **Almost never**  **1** | **Sometimes**  **2** | **Fairly often**  **3** | **Very often**  **4** |
| --- | --- | --- | --- | --- | --- |
| …been upset because of something that happened unexpectedly? |  |  |  |  |  |
| …felt that you were unable to control the important things in your life? |  |  |  |  |  |
| …felt nervous and stressed? |  |  |  |  |  |
| …felt confident about your ability to handle your personal problems? |  |  |  |  |  |
| …felt that things were going your way? |  |  |  |  |  |
| …found that you could not cope with all the things that you had to do? |  |  |  |  |  |
| …been able to control irritations in your life? |  |  |  |  |  |
| … felt that you were on top of things? |  |  |  |  |  |
| …been angered because of things that happened that were outside of your control? |  |  |  |  |  |
| …felt difficulties were piling up so high that you could not overcome them? |  |  |  |  |  |

**Below is a referral list of counseling services that can assist you, if you are experiencing any psychological distress that is affecting your mental health.**

1. AUB Counseling Center & AUBMC Psychiatry Department:

If you are a student at the American University of Beirut, please feel free to contact the Counseling Center at AUB West Hall, which provides free counseling services. Their number is 01-350 000 ext.

3196. You can also contact the Family Medicine Clinic (ext. 3000) or the Department of Psychiatry at AUBMC (ext. 5650) for appointments that qualify for student HIP-coverage.

1. Embrace:

Non-Profit Organization dedicated to Mental Health in Lebanon

-National Lifeline: 1564

-Mental Health Center: +96181003870

Email Address: info@embracefund.org

Address: Beirut, Lebanon

1. IDRAAC:

-Website: idraac.org

-Number: +961 3 730475

-Address: Achrafieh, St. George Hospital Street

Department of Nutrition and Food Sciences | Faculty of Agricultural and Food Sciences |AUB| Beirut, Lebanon

**Tips on how to cope with stress:**

- **Time management:** Use a planner to organize your schedule, and prioritize tasks.
- **Self-care:** Get enough sleep, balanced diet, regular exercise.
- **Social support:** Connect with friends and family regularly.
- **Set realistic goals:** Break down long-term goals into smaller, achievable steps. And celebrate your accomplishments.
- **Time for hobbies and relaxation:** Engage in activities you enjoy.
- **Study techniques:** Use effective study techniques.
- **Seek help:** If stress becomes overwhelming, don't hesitate to seek support from a counselor or therapist.
- **Stay informed:** Be aware of campus resources and support services available to students.

**Information about activities available at AUB:**

- **Sports facilities:** Tennis courts, gym, swimming pool, football field and many more.
- **Clubs:** Book club, Chess Club, Robotics Club, Art Clubs and many more.
- **Student societies**
- **Student governments**
- **Volunteer and community services**
